# An evidence-based primary health care intervention to address domestic violence against women in Brazil: a mixed method evaluation

**DOI:** 10.1186/s12875-023-02150-1

**Published:** 2023-09-25

**Authors:** Loraine J. Bacchus, Ana Flávia Pires Lucas d’Oliveira, Stephanie Pereira, Lilia Blima Schraiber, Janaina Marques de Aguiar, Cecilia Guida Vieira Graglia, Renata Granusso Bonin, Gene Feder, Manuela Colombini

**Affiliations:** 1 Department of Global Health and Development, London School of Hygiene & Tropical Medicine, Faculty of Public Health & Policy, 15-17 Tavistock Place, London, WC1H 9SH UK; 2 Preventive Medicine Department, Faculty of Medicine, University of São Paulo, Av. Dr. Arnaldo, 455 Cerqueira César, 01246 903 São Paulo, Brasil; 3 Population Health Sciences, University of Bristol, Canynge Hall, 39 Whatley Road, Bristol, BS8 2PS UK

**Keywords:** Domestic violence, Gender based violence, Primary health care, Health service evaluation, Health care providers

## Abstract

**Background:**

Health systems have a critical role in a multi-sectoral response to domestic violence against women (DVAW). However, the evidence on interventions is skewed towards high income countries, and evidence based interventions are not easily transferred to low-and middle-income countries (LMIC) where significant social, cultural and economic differences exist. We evaluated feasibility and acceptability of implementation of an intervention (HERA—Healthcare Responding to Violence and Abuse) to improve the response to DVAW in two primary health care clinics (PHC) in Brazil.

**Methods:**

The study design is a mixed method process and outcome evaluation, based on training attendance records, semi-structured interviews (with 13 Primary Health Care (PHC) providers, two clinic directors and two women who disclosed domestic violence), and identification and referral data from the Brazilian Epidemiological Surveillance System (SINAN).

**Results:**

HERA was feasible and acceptable to women and PHC providers, increased providers’ readiness to identify DVAW and diversified referrals outside the health system. The training enhanced the confidence and skills of PHC providers to ask directly about violence and respond to women’s disclosures using a women centred, gender and human rights perspective. PHC providers felt safe and supported when dealing with DVAW because HERA emphasised clear roles and collective action within the clinical team. A number of challenges affected implementation including: differential managerial support for the Núcleo de Prevenção da Violência (Violence Prevention Nucleus—NPV) relating to the allocation of resources, monitoring progress and giving feedback; a lack of higher level institutional endorsement prioritising DVAW work; staff turnover; a lack of feedback from external support services to PHC clinics regarding DVAW cases; and inconsistent practices regarding documentation of DVAW.

**Conclusion:**

Training should be accompanied by system-wide institutional change including active (as opposed to passive) management support, allocation of resources to support roles within the NPV, locally adapted protocols and guidelines, monitoring progress and feedback. Communication and coordination with external support services and documentation systems are crucial and need improvement. DVAW should be prioritised within leadership and governance structures, for example, by including DVAW work as a specific commissioning goal.

**Supplementary Information:**

The online version contains supplementary material available at 10.1186/s12875-023-02150-1.

## Background

Intimate partner violence (IPV) is a major public health problem, damaging the health of women and their families [1–5]. It affects 1 in 3 women globally [6] and 45.3% of women using primary health care clinics in the metropolitan region of São Paulo (SP), Brazil [7]. Health systems have a critical role in a multi-sectoral response to IPV [8], but the evidence on interventions is skewed towards high income countries (HIC), and lessons learned are not easily transferred to low-and middle-income countries (LMIC) due to social, cultural and economic differences [8].

In HIC, there is growing evidence that interventions to improve PHC responses to DVAW can raise identification and referrals to external services [9], connect women to multi-agency networks and also improve depressive symptoms [10]. Whilst there are examples of promising interventions from LMIC that reduce re-exposure to some forms of VAW and improve some health outcomes, the evidence is limited and mixed [11–13]. Evaluations focus on individual provider and clinic level factors that affect implementation rather than systemic strengthening of capacity within the health system [14]. This is the first Brazilian study to develop and evaluate an intervention to improve the PHC response to domestic violence against women (DVAW).

In the past two decades, Brazil has advanced in including sexual and domestic violence in its National women's health policy, created a domestic violence law (Maria da Penha law) [15] and expanded a specialised network to assist VAW cases. PHC providers recognise their crucial role in addressing DVAW, but they lack guidelines, clearly defined roles and care pathways  to support them in responding to DVAW and its complexities [16].

Our study was conducted in municipal PHC clinics in (SP), a city of over 12 million people in Brazil. Family health teams in the clinics consist of a doctor, a nurse, two nursing assistants and six community health workers (CHW) responsible for 800—1000 families [17, 18]. In SP, the PHC clinics are managed by Social Organizations (Organizações sociais—OS), private not-for-profit organisations through contractual agreements with the National Health System (SUS) to deliver performance targets focused on maximising the number of consultations [19].

In SP city, since 2002, there has been growing interest in raising awareness of the role of health services in dealing with violence, including DVAW [20] [21–24]. However, despite numerous guidelines published by the municipal secretary of health, they tend to be vague, not widely recognised and are infrequently read by health care workers. Additionally, there is a lack of continuity in the implementation of these policies dues to changes in leadership among mayors and health secretaries [20, 25]. In 2015, a Violence Prevention Nucleus (NPV) was established as mandatory in every SP health service as part of the municipal care pathway policy to violence, as part of a broader policy framework developed by the municipal secretary of health [26]. The NPV is responsible for training, epidemiological surveillance, coordinating support for cases of all forms of violence and referral to multi-agency networks. Despite a comprehensive policy, the implementation of a DVAW response in the primary health care system has been weak [20, 25].

We conceptualised the health system as a complex adaptive system in which interaction between individual agents (e.g. providers, patients, policy makers, communities) leads to continually emerging and novel behaviour [27]. This paper presents findings from the first Brazilian study evaluating an intervention aimed at strengthening the PHC response to DVAW. The study was conducted in collaboration with a global health group known as Healthcare Responding to Violence and Abuse (HERA), which included partners from the UK, occupied Palestinian territories, Nepal and Sri Lanka. The evaluation explored factors that shaped translation of the intervention model into activities, assessed feasibility, acceptability and changes in identification of DVAW and referral to support.

## Methods

The research used mixed method process and outcome evaluation with a six-month follow-up. Following implementation of HERA, this study aimed to assess: (i) feasibility of the intervention; (ii) changes in  PHC providers readiness to identify, document and respond to DVAW; (iii) PHC providers' perceptions of their personal safety and support received to deal with DVAW; (iv) whether clear roles within the clinical team (and mutual appreciation of them) promoted collective action in the coordination of DVAW cases within and outside of the health system; (v) the acceptability of the intervention to women and its success in meeting their support needs.

### Brazilian HERA intervention development and theory of change

The HERA generic intervention model provides training and supervision to support PHC providers in addressing DVAW. In Brazil, the intervention development was informed by formative research [25] that aimed to analyse the health system's readiness to integrate response to DVAW, mapping obstacles and facilitators. Intervention development also drew on a UK evidence-based domestic violence intervention for primary care[9], the Brazilian model of Confad (Difficult Family Conflicts) [28] and the WHO clinical and policy guidelines [29, 30].

**Fig. 1:**
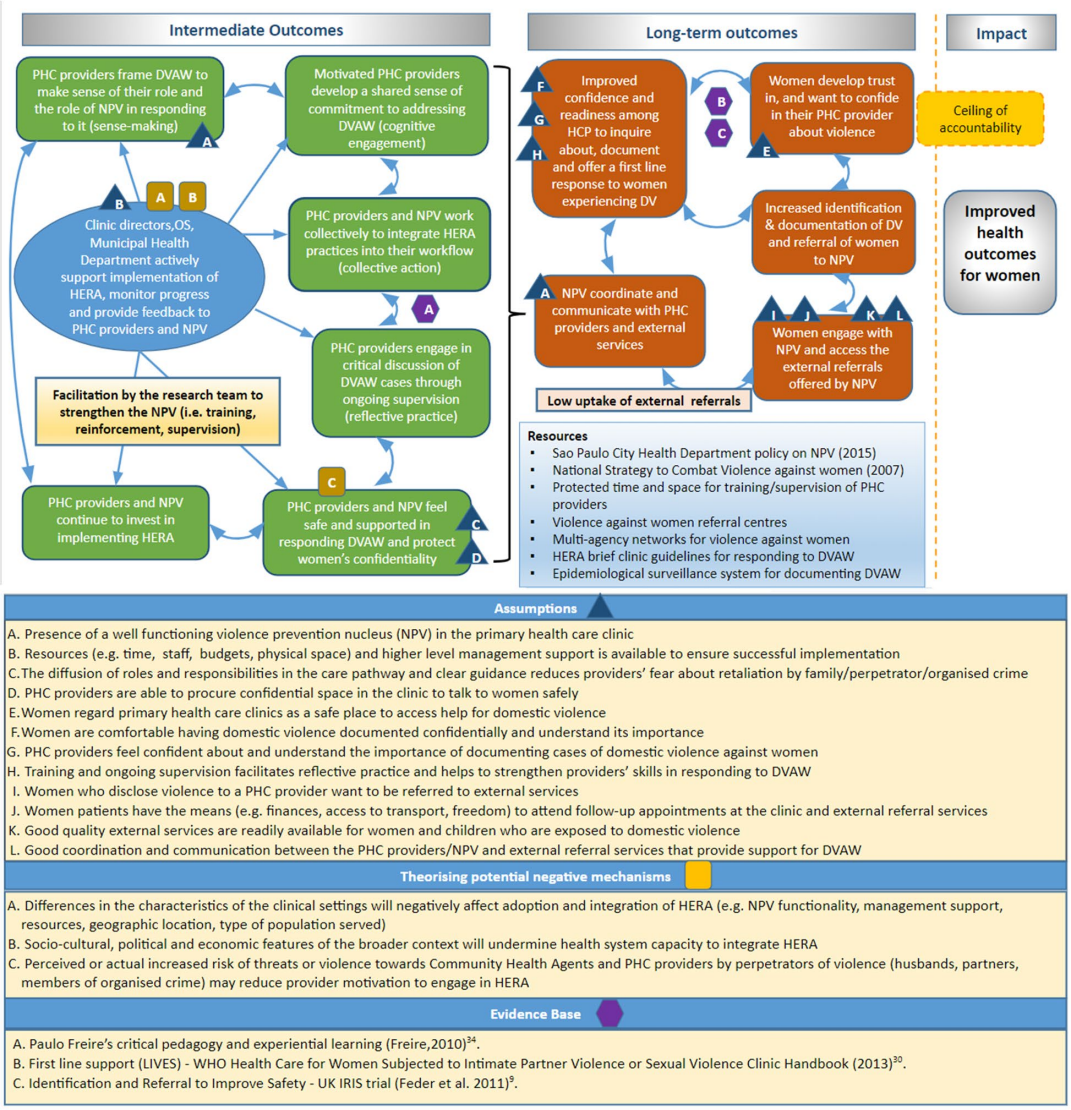
Theory of Change. PHC—Primary Health Care; DVAW—Domestic Violence Against Women; NPV – Núcleo de Prevenção da Violência (Violence Prevention Nucleus); OS – Social Organization

A theory of change (ToC) was developed (Fig. 1), which surfaced mechanisms about how the intervention was expected to work at different stages (intermediate outcomes), and the conditions required to enable change (assumptions), in order to achieve the desired longer-term outcomes [31, 32]. We hypothesised negative mechanisms (‘dark logic models’) that could potentially undermine implementation or cause adverse outcomes for those delivering or receiving the intervention [33]. The ceiling of accountability separates the future impact (i.e. improving health outcomes for women) that is considered outside of the sphere of influence of the HERA intervention and therefore not monitored by the evaluation (Fig. 1).

### Description of the Brazilian HERA intervention

**Fig. 2:**
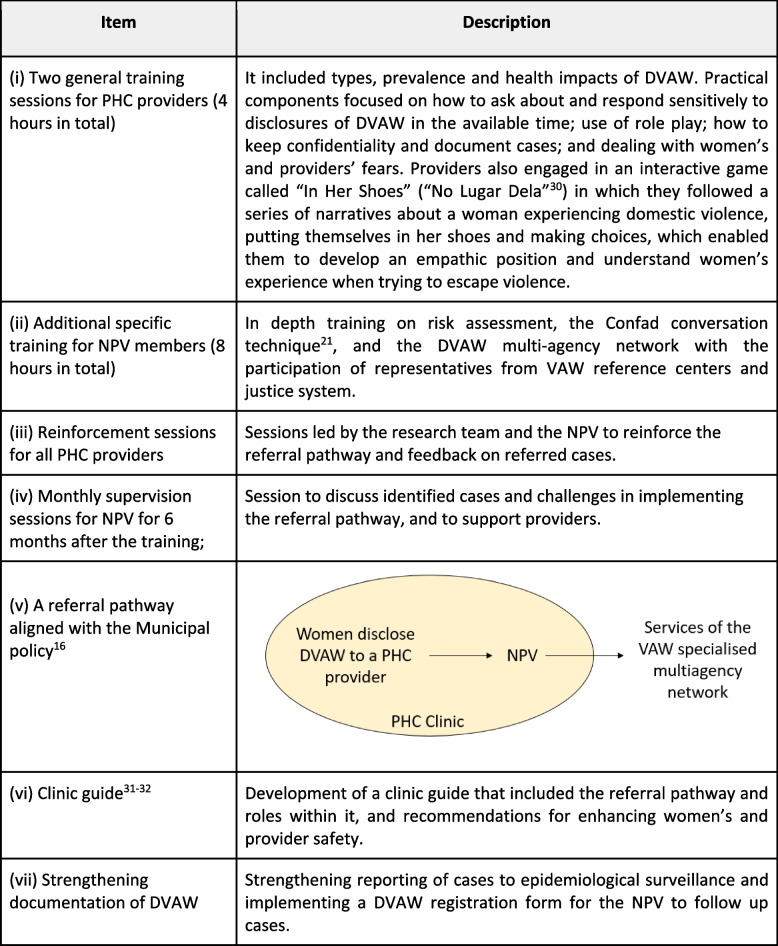
Components of HERA intervention in Brazil

The components of the intervention were developed by the research team in consultation with key stakeholders (Fig. 2). We delivered a four-hour in service training between April and May 2018, organised in two sessions of two hours with a one month gap between sessions. A total of 16 sessions were delivered to train all PHC providers in the two health clinics. We worked in smaller groups to avoid disruptions to  the health service. A first line response (LIVES) was provided to women who disclosed violence [30]. All PHC providers were trained to listen (L), inquire (I) and validate (V), whilst the NPV members were given additional training to enhance safety (E) and support (S), thus dividing the responsibility to accommodate the demands of a busy clinic environment. Drawing on Paulo Freire's critical pedagogy [34], participants engaged in critical reflection through a gender and human rights lens.

Training to PHC providers was delivered by the researchers, who have expertise in delivering training on DVAW. NPV were responsible for dealing with DVAW cases referred to them and supporting PHC providers. However, our formative research revealed that the assumption about a functional NPV in each clinic was incorrect (Assumption A, Fig. 1). It was necessary for the research team to strengthen the NPV as *facilitators* within the intervention [35]. Therefore, additional training was offered to the NPV members in June 2018. This paper used the reporting standards of the template for intervention description and replication (TIDieR) checklist and guide [36] (Additional file 1).

All numbered references [26, 28, 37–39] in the figure are cited at the end of the paper. The protocols developed by the research team are available to download in the citations.

### Setting and sampling

The research was conducted in two PHC clinics in SP selected by regional managers as "typical" (i.e. not exceptionally good or bad), with Clinic 1 serving a more socioeconomically deprived area [25]. PHC providers were purposely sampled by diversity of age, profession, and willingness to engage in DVAW response as perceived by the clinic director. Women who disclosed domestic violence after implementation of HERA were informed about the study by their PHC provider and consent for contact sought by researchers. All participants who agreed to participate in the study were contacted by the researchers via telephone to schedule the interview. The number of interviews were defined by saturation criteria, except for the interviews with women, as it was challenging to recruit and interview survivors. Notwithstanding, we included the women's interviews, because their perceptions and experiences offered valuable insights about how to improve interventions for addressing DVAW in PHC services.

### Data collection

Between October 2018 and January 2019 (six months after training), 17 semi-structured face-to-face interviews were conducted with 13 PHC providers, two clinic directors and two women who disclosed domestic violence (Table 1). All providers attended the training, other than a social worker, a key worker within the NPV. Interviews were conducted in Portuguese by trained female researchers: AF and JMA (PhD); SP (MSc); CGVG and RGB (BSc), ensuring that the researcher had not delivered training to the participant. The interviewers had backgrounds in medicine, nursing and psychology, in addition to previous experience of qualitative research.

**Table 1:** Characteristics of participants

**Study Participants**	**Features**
**Women**	***n* = 2**
**Clinic**	
1	1
2	1
**Ages**	
Woman 1	45
Woman 2	72
**Length of marriage/relationship**	
Woman 1	3 years
Woman 2	25 years
**Number of children**	
Woman 1	1
Woman 2	3
**Education**	
Woman 1	high school
Woman 2	high school
**In paid employment**	
Woman 1	Housewife (sick to work)
Woman 2	Cleaner
**Domestic Violence Perpetrator**	
Woman 1	Intimate partner
Woman 2	Daughter
**Access to the NPV**	
Woman 1	Poster in the clinic
Woman 2	Referred by a doctor
**Healthcare providers**	***n* = 15**
Clinic 1	8
Clinic 2	7
**Age range**	28 to 70
**Sex**	
Female	13
Male	2
**Job title**	
Physician	2
Nurse	2
Nurse technician	2
Social worker	3
Psychologist	1
Clinic director	2
Community health agent	3
**NPV**	
Yes	4
No	11

The interviews were carried out in a room in the PHC clinic, ensuring privacy for the researcher and the participant. The interview topic guides were developed by the research team and pilot tested among PHC providers of clinics that were not part of the study. The interview guide for PHC providers included questions about the training and how well it prepared them to deal with DVAW; individual, clinic and system level challenges during implementation; and perceptions of how the referral pathway worked and their roles within it. The women’s interview guide included questions about their experiences of talking to PHC providers at the clinic and the support they were offered and accessed. The median duration of the interviews were 47 min, ranging from 26 – 78 min.

We assessed participation in the training through attendance records. Anonymised data on identification of DVAW and referral were obtained from the Municipal Health Department which collates clinic level data on violence reports for the Epidemiological Surveillance System (SINAN) [40]. The epidemiological surveillance database was checked for consistency and we selected all registered cases of psychological/emotional, moral, physical, economic and sexual violence experienced by women aged 18 years and older, reported 12 months before (May 2017—April 2018) and after (May 2018—April 2019) the intervention. The database included socio-demographic information and referrals to services of the multiagency network. Additionally, we tracked the referral uptake directly at the DVAW centres.

### Reflexivity

The dual role of the research team as evaluators and facilitators of the intervention required some caution, but created the advantage of being organically situated in the field. Critical reflection regarding our own perspective and position was intricately woven into field work and analysis. The complex role of insider/outsider that we occupied required a conscious effort to understand the "other" (i.e. health care providers) whose world view was both familiar to us, but also unknown [41].

### Data analysis

Interviews were audio recorded and transcribed verbatim into Portuguese. Six interviews and a report of the analysis was translated into English to facilitate data analysis workshops with UK collaborators. All authors read and annotated the same set of interviews separately followed by discussions to identify recurring patterns and unusual events or accounts, using thematic content analysis [42]. We developed and refined a coding frame with additional interviews. Coding was deductive, drawing on key areas in the topic guides and inductive to allow new themes to emerge. NVivo11 was used to facilitate coding and data organisation. Descriptive analysis was undertaken using numbers and proportions for training attendance, identification of DVAW and referral data.

### Ethics

Ethical approval was obtained from the University of São Paulo (2.056.530), University of Bristol (61603) and the London School of Hygiene & Tropical Medicine (15341). Researchers had a referral pathway for women, patients of the clinic or PHC providers, in need of immediate support.

## Results

The findings are presented around five key themes that respond to our research questions. Table 1 presents the sample characteristics.

### Feasibility of the intervention

#### Delivery of training

In total, 99 (51.0%) PHC providers from the two clinics attended training (Table 2) and 15 NPV members participated in additional training, including providers that showed interest in joining the NPV after the PHC training.

**Table 2:** PHC providers trained in each clinic

**Number of sessions attended**	**Clinic 1**			**Clinic 2**		
Both training sessions	34			20		
First session only	20			14		
Second session only	5			6		
**Total**	59 (of 72)			40 (of 107)		
**Percentage of clinic staff**	82%			37%		
**Type of provider**	**Trained**	**Total**	**%**	**Trained**	**Total**	**%**
College graduate	17	23	74	20	52	38
Technician (e.g. nurse technician, dentistry assistant)	17	18	94	0	17	0
Lay workers	25	31	81	20	38	53
**Sex**	**Trained**	**Total**	**%**	**Trained**	**Total**	**%**
Female	45	56	80	32	82	39
Male	14	16	88	8	25	32

OS managers interrupted a training session at Clinic 1 and allocated half of the PHC providers to another task, despite a formal agreement to support the intervention. Training activities were hampered and rescheduled because of the measles outbreak and accompanying vaccination campaign, when all training activities in the municipal health clinics were suspended for three months. This suggests that domestic violence work and training in general were lower priority and exacerbated by staff shortages (Assumption B, Fig. 1).

The research team experienced barriers to conducting reinforcement training sessions as Clinic 1 did not have regular team meetings. Clinic 2 had monthly team meetings, and we conducted two brief sessions plus one reinforcement session enabled by the clinic director.

Providers stressed the need for periodic reinforcement training to strengthen skills and address the issue of staff turnover which could erode long-term sustainability of HERA.

*There is a turnover of professionals… They had professionals who were borrowed, who were from another unit, and doctors who were leaving and others who entered. So today we have professionals who are super interested, who want, but who were not at the time here, understood?* [Social Worker, NPV, Clinic 1]

### Impact on provider readiness

#### Developing competencies: “Widening the gaze” beyond the medical model

The increased interest in and visibility of DVAW engendered new understandings and reflections about the limits of the traditional biomedical model for responding to DVAW. Providers’ perceptions of their role evolved from a more passive stance prior to training that expected women to speak out, to a more active one after the training—“*I must and I can be interested*” and “*Everyone should be interested*” [Doctor, Clinic 1]. This sense-making process helped “to *widen the gaze”* [Social Worker, Clinic 1] of providers to the DVAW context and how it related to symptom presentation, emotional distress, and clinical diagnoses (foremost in the causal chain of intermediate outcomes). Supervision meetings were important spaces to engage providers in critical discussion. Consequently, the repertoire of provider actions broadened, and responses such as welcoming, listening, supporting, providing care, and avoiding moral judgement of women's accounts, were more valued by PHC providers after training.

*It has greatly improved the way we look, in cases of violence, you have a neutral gaze too, without judgement … I think that was a crucial point … If you begin to judge, the person will shut down and will not be open with us anymore*. [Community Health Worker, Clinic 2]

Providers valued a training exercise which placed them in the role of an abused woman negotiating multiple obstacles. This approach encouraged the deconstruction of moral judgements regarding violence against women and helped providers to reflect upon the potential repercussions of their actions or inaction. It also promoted a woman-centred and gendered response.

*When it comes to the … real life stories we played … where you could modify that story. We start to think … that we could do things differently … You think “I had another choice”. I think it stirred a lot with everyone who participated*. *I found it cool and very practical as [in] what to ask, what to say. Because I think this often makes the professionals stay away from these types of cases.* [Nurse, Clinic 1]

Training increased providers’ confidence and skills in identifying and responding to DVAW and they were more attentive and sensitive to behavioural cues consistent with experiences of abuse. For example, linking signs such as crying, stress and the presence of a controlling partner with a greater likelihood of DVAW. Providers began to question some relationship behaviours (e.g. verbal abuse) which they had previously regarded as normal and were able to *read between the lines* of women’s accounts.

Securing confidential space with women did not appear as a barrier to dealing with DVAW cases before the intervention, but emerged as a challenge after because providers were identifying more cases and attuned to the need for privacy. Providers found some solutions such as using rooms with greater privacy or placing “In session, please wait” signs on consulting room doors to avoid interruptions (Assumption D, Fig. 1).

#### Identification, referral and documentation of domestic violence cases

Lack of time to attend to patients experiencing DVAW was less present in provider narratives after training. This may be attributed to HERA, which emphasised teamwork, confidentiality and the internal referral pathway. This gave providers a way to respond effectively in the available time and increased their comfort in asking about violence. This was corroborated with data on identification of cases in both clinics, as well as referrals, both to the NPV and the external specialised network.

*... it was not that there was an increase in demand. I think there was an increase in the perception of the cases.* [Physician, Family Health Strategy Team, Clinic 2]

**Fig. 3:**
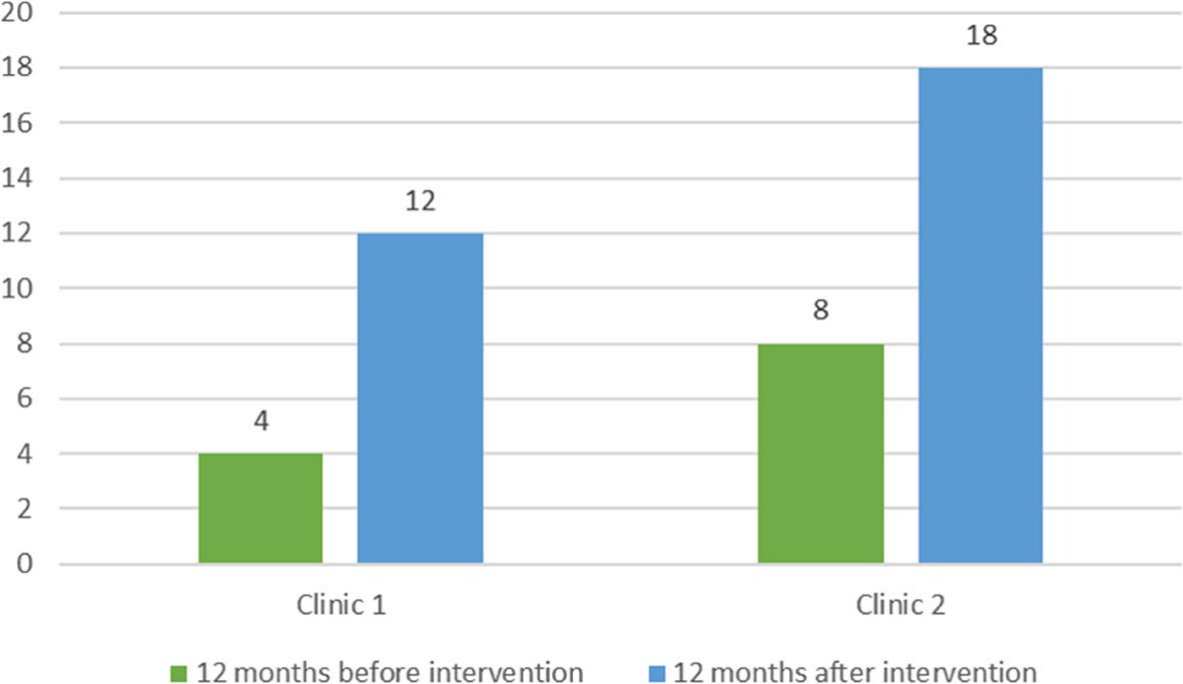
Number of DVAW cases reported to the mandatory epidemiological surveillance before and after HERA intervention. Source: SINAN—Violência. Access on 20/01/2020. TabNetSMS/COVISA/DVE/NDANT. This graph only includes DVAW cases of women over 18 years old. Timeline: Pre-Intervention—May 2017 to April 2018; Post Intervention—May 2018 to April 2019. DVAW = Domestic violence against women

Fig. 3 shows the number of DVAW cases aged 18 years and over reported to the mandatory Epidemiological Surveillance System 12 months prior to and after HERA. There was an increase in both clinics. The paper-based system was perceived as cumbersome, complicated and time consuming to complete. There was also a lack of clarity regarding its purpose and whose responsibility it was to complete it, although it appeared that NPV were doing this.

Providers spoke about informal documentation systems which they developed to track the evolution of cases and their involvement in them. Some CHAs used personal notebooks and used the term ‘family conflict’ to indicate the presence of DVAW. This was sometimes used to give feedback about families during team meetings. NPV kept their own records which focused on the violence experienced and external referrals offered, but they did not keep records of cases which were not referred to them. The multiple sources of documentation demonstrated its importance to providers, but it created extra work and confusion between official and personal records, leading to lack of coordination and possible loss of information (Assumption G, Fig. 1).

### Enhanced feelings of safety among providers and women

The existence of a clinic guideline combined with clear roles and a referral pathway appeared to enhance feelings of safety among providers and comfort in dealing with DVAW (Assumption C, Fig. 1). Before the intervention, providers’ fears centred on how their actions could worsen the situation and place women and themselves at risk of retaliation from the perpetrator. Fear was described as something that paralyses providers, sometimes causing them to turn a blind eye.

Personal safety, confidentiality and concrete strategies to ensure safe practices were extensively covered in the training. In addition, the clinic guidebook created for HERA included a section on how to prevent risks to the woman and providers. There was no evidence of perceived or actual threats of violence towards providers leading to reduced motivation to engage in HERA (negative mechanism C, Fig. 1). The impact of these actions was present in the views of providers, clinic directors and women.

*Because we get better instrumentalized, I think that it comes from the proper approach … and what I have as a support tool, so fear diminishes … You are often afraid of what you do not know. When you get to know what you have available, whether it be equipment, instrument or team, fear decreases.* [Nurse, Clinic 2,]

The diffusion of responsibility for responding to DVAW and particularly the key role of NPV was also a factor which enhanced perceptions of safety. Collective action meant that no single provider could be linked to a case, and the intervention agreement was that any formal report to other agencies should be signed by the clinic directors, in the name of the whole team.

### Mutual appreciation of roles to promote collective action

#### Differential management support and leadership

There were differences between the two clinics with regards to management style and engagement with consequences for implementation. Clinic 2 had a more stable and effective management. The director in Clinic 2 was more proactive in organising the training schedule and releasing providers from clinics to attend training. Even though Clinic 2 had a lower percentage of trained PHC providers, it included more providers educated to university level (doctors, nurses, psychologists, social workers). In practice, they had more autonomy over agendas and influenced the team to be more sensitive and attuned to DVAW cases. The director from Clinic 2 allocated space for meetings with all the clinic providers, which helped to implement the internal flow for the identified cases. The NPV meetings were protected in the agendas of the members and therefore, the supervision sessions to discuss the cases in those spaces. The Clinic 2 director also assisted providers with domestic violence cases and referrals to the inter-sectoral networks.

*From the beginning, the director of the clinic always supported us, and even participated in some cases guiding us … If we did not have the support of the director, even more regarding the NPV … we would not have the same strength, right?* [Social Worker 1, NPV, Clinic 2]

However, clinic directors and PHC providers conceptualised the director's role as not obstructing the actions of providers, helping individuals with difficult cases and responding to urgent demands, as opposed to actively implementing and monitoring the NPV policy. Thus, there was some indication that differences in the characteristics and organisation of the clinical settings affected integration of HERA (negative mechanism A, Fig. 1).

#### Strengthening the NPV to promote collective action

HERA increased awareness of and strengthened the NPV. In both clinics new PHC providers were included in the NPV, meetings occurred more frequently, their range of activities broadened to include assistance to women and multi-agency network contacts, all of which enhanced the internal recognition and value of NPV. Before the intervention, Clinic 1 had one NPV member and added six CHW and three assistant nurses to it as a result of HERA, whilst Clinic 2 had two NPV members and added three social workers and psychologists to it.

After the training, the referral pathway was assimilated by many providers as it offered a tool that guided their actions. They described feelings of trust in the NPV which appeared to increase their comfort in identifying DVAW cases. From the identified cases, 9 in Clinic 1, and 18 in Clinic 2 were referred and attended by the NPV.

*Because in a situation like this, what if I did not have NPV? I would take this situation to whom? ... So actually the NPV for me is a tool … I did not know that there was such work. You say, "Wait. I'm not alone."* [Community Health Worker, Clinic 1]

HERA strengthened the clinical team response to DVAW, where providers developed a shared commitment to the intervention and an appreciation of each other’s roles and responsibilities. Their accounts pointed to the complementarity of these roles and the need for collective action. Domestic violence was no longer perceived as a one-dimensional issue (e.g. a mental health issue) and therefore the domain of a single professional (e.g. psychologists).

*So, thinking of a multi-professional team… but also the administrative… [staff], paying attention to these situations, I think it greatly facilitates the work of the clinic as a whole … The majority ...“See, they have the NPV. Would you like to speak to them?” Then they send them to us. So it's a flow that's working fine.* [Social Worker 1, NPV, Clinic 2]

Table 3 shows that external referrals were made to a range of services by the NPV due to their increased awareness of the multi-agency network after training.

*That there are places that people get help, which was something I also did not know. That there is a place they can go to, there is a shelter, and these 'things' … We always thought the woman had to find a place by herself. The* (training team) *showed you various ways*. [Nurse Technician, Clinic 1]

**Table 3:** Referrals of DVAW cases reported to the mandatory epidemiological surveillance before and after intervention

**External referral offered**	**Clinic 1**				**Clinic 2**			
	**Before**		**After**		**Before**		**After**	
	**n**	**%**	**n**	**%**	**n**	**%**	**n**	**%**
Health sector	3	38	7	30	6	40	12	36
Social assistance	1	13	7	30	1	7	3	9
DVAW Reference Centre	3	38	7	30	4	27	12	36
Police station	1	13	2	9	4	27	6	18
**Total**	**8**	**100**	**23**	**100**	**15**	**100**	**33**	**100**

Source: SINAN—Violência. Access on 20/01/2020. TabNetSMS/COVISA/DVE/NDANT

This table only includes DVAW cases of women over 18 years old. Women could be referred to more than one service. Timeline: Pre-Intervention—May 2017 to April 2018; Post Intervention—May 2018 to April 2019

*DVAW* Domestic violence against women

We tracked the external referrals made to the DVAW reference centre after the training. In Clinic 1, two women took up the referral. In Clinic 2, one had already accessed the centre and three additional women took up the referral.

Whilst improved knowledge of services available to women was seen as contributing to the increased rates of identification, providers highlighted the poor availability and/or quality of services. Providers’ distrust of external referral services was considerable and linked to their unrealistic expectations or negative experiences of referral in the past. The assumptions about women wanting to be referred to external services and the quality of services available were not borne out in reality (Assumptions I to K, Fig. 1).

*Sometimes we also have a false impression that ... look, the facility, it will save the day! It will get a shelter, it will get the woman out of that situation, it will find a way. You know? And it's not always like this! ... We do not know about their difficulties, and they do not know about ours … This is a lack of dialogue.* [Social Worker 1, NPV, Clinic 2]

After the training, the communication between the clinics and DVAW services improved through meetings, case discussions, and even a few consultations conducted by DVAW service providers at the health clinics for women who were unable to go to the DVAW centre (Assumption L, Fig. 1). Although not an expectation, NPV went beyond their role and preferred to make assisted referrals, accompanied women to services, organised transportation and checked that women had arrived at the service. This emergent feature of the care pathway demonstrates the motivation of the NPV and their investment in HERA. They tried to liaise with services in a more organised way to obtain feedback on women, but turnover of providers and the precarity of the DVAW service in a time of disinvestment in the DVAW network made it challenging, as described by one woman:

*The social worker left [the VAW reference centre]. I had one appointment with her and she left … another one replaced her and I have not yet met her. The psychologist too. I had two appointments with her and she also left. After Bolsonaro [Brazil’s president at the time] won, she left. There is also the lawyer [of the VAW reference centre], right? We talked about me leaving the house and about alimony, these sort of things. She advised me to reach out to the justice system to get my house back… And then they all left, and I do not have any contact with the new staff. *[Survivor, Clinic 1]

In general providers acknowledged the need for improved coordination both between and within services.

### Women’s views on acceptability of HERA

Women described their interactions with PHC providers as empowering and encouraging, relating a delicate process of listening and advising. Prior to HERA, women disclosed violence but were offered antidepressants and tranquillisers with no specific advice. Following the training, women’s decisions were more respected which allowed self-transformation at their own pace.

*The doctor prescribed me Clonazepam, then gave me Fluoxetine, that also did not help much. The health providers here understood me. They understand that I did not want to go to the shelter, and that I need someone to listen, to help me. They respected me. They never told me: "this is better for you" or "you are wrong in that". They allowed me to make my own decision … I felt much safer than if I was talking with a family member or friend.* [Woman]

Women valued the opportunity to talk about DVAW with a provider, and both women interviewed reported that they had more trust in their PHC provider’s respect for confidentiality, compared with talking to friends and family (Assumption E, Fig. 1). This reflects women’s fear of stigma and judgement in the community. Regardless of which type of provider women had contact with, they were all praised for their supportive and consistent team approach.

## Discussion

HERA seemed to be feasible and acceptable to women and PHC providers. The findings suggest it has potential to improve providers’ skills in identifying and responding to DVAW within a woman-centred, gender and human rights perspective. Integrating gender and power inequalities contributing to DVAW into the training was critical to providers’ reflecting on their own contributions regarding the lack of respectful and potentially re-traumatising care to survivors [43]. HERA helped to diversify referrals outside the health system and strengthened the NPV. PHC providers felt safe and more supported due to the clear roles, collective action within the clinical team, guidelines and management support.

The ToC helped to identify weak causal pathways, such as poor implementation of the NPV policy, low uptake of external referrals, intrinsic contextual factors and mechanisms that contributed to implementation success. Some of the ToC intermediate outcomes reflect core constructs within Normalisation Process Theory [44]; that of coherence (how providers made sense of HERA and its possibilities), cognitive participation (commitment to HERA, and shared values and practices that gave legitimacy to the work), collective action (how providers mobilised skills and resources to make HERA workable) and reflexive monitoring (appraising the effects of the intervention). The intermediate outcomes in ToC were responsible for shaping the actions of providers and formed a feedback loop with higher level management support at the centre.

It was challenging to train all staff due to the busy clinic environment and competing productivity goals. Although attrition during training was high, HERA seemed to contribute to an improved sensitivity of HCP towards DVAW. Participation in supervision was better in Clinic 2 than Clinic 1, probably due to the stronger support of the local and regional manager who had oversight for supporting the NPV policy in all health services in the region. An evaluation of HERA in Palestine also found that high-level support within the health system is needed to legitimise the role of providers in responding to DVAW [45].

A key finding was the importance of management support in implementing HERA. Our formative phase research which explored the readiness of the health system to integrate HERA in Brazil, found that regional managers and clinic directors did not have clear roles in supporting DVAW work and the policies were loose and unclear [25]. We strengthened this aspect of readiness to integrate HERA by developing a care pathway with clearly defined roles, standard operating procedures, and the inclusion of clinic directors in DVAW training. However, we could not mitigate the effect of the OS performance management model, which prioritised more, shorter consultations. OS is associated with more precarious working conditions, lack of engagement with the community and an authoritarian management style [46]. The literature shows how some contexts are more conducive to successful implementation than others, due to the presence of transformational leaders, features of learning organisations, appropriate monitoring, and evaluative feedback mechanisms [47]. Committed directors can promote implementation through diffusing information; mediating between strategy and daily activities; and ‘selling evidenced-based implementation’. The implementation climate (i.e. practitioners’ collective perception that evidenced-based implementation is rewarded, supported and expected within an organisation) can mediate between these managerial functions and implementation success [48].

PHC providers developed a new understanding of their role. There was a shift in values and practices, which was evident in providers' recognition of the limits of the medical model and incorporating new skills. PHC providers placed more importance on providing "care" (understood as responding to the broader needs of women and recognising the social determinants of health), as opposed to "treatment" (understood as the restrictive biomedical model of diagnosis and treatment of disease). As a result of this sense making, they were less judgmental and respectful of women's choices, and most of them rejected the normalisation of violence and victim blaming. Furthermore, they valued and looked for evidence of “practical success” (that reinforced the PHC providers’ and women’s experiences and voices) as opposed to only “technical efficacy” (defined by biomedical knowledge) [49]. The fact that HERA was developed on SUS principles (i.e. comprehensive and person-centred care, being welcoming and bonding with patients) contributed to these outcomes. SUS principles are deeply embedded in Latin American Social Medicine which recognises the social determinants of health [50]. However, the findings should also be understood within the political and economic context of Brazil. Structural problems persist in SUS, including gaps in organisation and governance, lack of public funding and suboptimal resource allocation. This has been exacerbated by austerity policies introduced by the current government, resulting in large regional and economic disparities in access to healthcare services and health outcomes [51].

Whilst these broader intervention-context interactions weakened implementation of HERA, many PHC providers continued to collectively invest in responding to DVAW. Given the sensitive and complex nature of DVAW [52], supervision sessions were paramount for supporting PHC providers to work with cases. Supervision provided technical guidance on case follow-up and emotional support to HCP in dealing with their own feelings while providing care to women. Although supervision and mentoring sessions have been highlighted as important intervention features to strengthen the health system response to the DVAW [8, 53], there is still incipient literature that discusses its role in the implementation and sustainability of interventions.

HERA strengthened the NPV by increasing their numbers and their value in supporting the clinic response to DVAW. The collective response to DVAW helped to alleviate PHC providers’ fear of retaliation and worsening the situation for the women as evidenced in other studies [54]. However, the assumption regarding the functionality of NPV was not borne out in reality and directors and NPV needed to be mentored in realising their role. This necessitated intervention by the research team who developed and delivered training to and supervised NPV in the coordination of complex DVAW cases through critical reflection and dialogue. This allowed them to co-create and model effective practices, thus helping NPV to bring about change within the clinical teams. This dual role as researchers and facilitators within the HERA intervention was an emergent feature of the implementation process. The research team enabled reflective learning, encouraged critical thinking, dealt with psychological defensiveness and challenged cultural norms about DVAW. Intervention facilitators can support implementation of changes in practice, helping individuals and teams to understand what they need to change and how they need to change it [35]. Further research is needed to explore how facilitation and mentoring can be used to support managers in prioritising and integrating DVAW work into clinical practice.

Documentation of DVAW was complex and providers used a range of formal and informal mechanisms for very different purposes. Although reporting to the surveillance system is mandatory, underreporting of domestic violence cases is common [55, 56].

Issues that discourage providers from documenting DVAW, include lack of time, and fear of retaliation [45, 57]. The agreement to have the signature of the clinical director on notifications of DVAW instilled trust among providers to notify the cases. The use of epidemiological notification data, despite underreporting, enabled the comparison of identification of DVAW before and after the intervention.

The two interviewed women found it acceptable to be asked about DVAW by a PHC provider and valued the confidentiality offered and non-judgemental approach. They appreciated the empathic and woman-centred care more than the offer of referrals to external services, a finding which has been documented in other studies from LMIC [45, 58, 59]. Women’s reluctance to engage with external services may be due, in part, to the close relationships they established with PHC providers to whom they disclosed violence and the continuity of care offered which fostered trust. Other factors include stigma, lack of time, human resources, transportation and availability of services.

### Strengths and limitations

The ToC facilitated the adaptation of an evidence-based intervention to the Brazilian context. The research team delivered training, which resulted in their dual role of insider/outsider. Whilst this permitted a deeper understanding of the implementation process, it is possible that they were less aware of their biases. Strategies to address this included critical reflection, field notes, and team discussions. The inclusion of the UK team as an "outsider" interlocutor during analysis facilitated this process. The evaluation captured differences in the administration and commissioning of services (i.e. from government to OS) at the clinics which impacted implementation. It was challenging to recruit women survivors from the clinics. Interviewing more women living in diverse circumstances would have generated a wider range of accounts regarding help-seeking. SINAN data are likely to be an underestimate due to the multiple documentation systems in place and inconsistent documentation practices reported by PHC providers. The six-month post-intervention follow up did not allow us to evaluate long term sustainability.

## Conclusion

The intervention helped to *widen the gaze* of PHC providers through a broader and gendered conceptualisation of health and its determinants, increased identification and broadened referral options. However, sensitisation and skills-based training of PHC providers on DVAW was not enough to ensure sustainable changes in practice. Training should be accompanied by system-wide institutional change including active (as opposed to passive) leadership and management, allocation of resources to support roles within the NPV, locally adapted protocols and guidelines, monitoring and feedback, and communication and coordination with external support services and local community [60]. The integration of evidence-based DVAW interventions into ‘real world’ settings requires continuous reflection and adaptation in order to embed and sustain new ways of working.

### Supplementary Information

**Additional file 1.** The TIDieR Checklist*: completed on the HERA intervention in Brazil.

## Data Availability

The datasets generated and/or analysed during the current study are not publicly available due sensitivity of the data but are available from the corresponding author on reasonable request.

## Abbreviations

CHW: Community Health Workers
DVAW: Domestic Violence Against Women
HCP: Healthcare providers
HERA: Healthcare Responding to Violence and Abuse (Global Health Group)
LMIC: Low and Middle Income Countries
NPV: Núcleo de Prevenção da Violência (Violence Prevention Nucleus)
OS: Organizações Sociais (Social Organizations)
PHC: Primary Health Care
SINAN: Brazilian Epidemiological Surveillance System
SP: São Paulo
SUS: Sistema Único de Saúde (Universal Health System from Brazil)
ToC: Theory of Change
UK: United Kingdom
