# Supplementary material for: An evidence-based primary health care intervention to address domestic violence against women in Brazil: a mixed method evaluation
Source: BMC Prim Care. 2023 Sep 25;24:198. doi: 10.1186/s12875-023-02150-1 (PMC10519067; doi:10.1186/s12875-023-02150-1)
Supplement: Supplementary file 1 — Additional file 1. The TIDieR Checklist*: completed on the HERA intervention in Brazil. [file 12875_2023_2150_MOESM1_ESM.docx]

COREQ (COnsolidated criteria for REporting Qualitative research) Checklist

| **No** | **Item** | **Guide questions/description** | | **Reported on Section/ page number/ paragraph** | |
| --- | --- | --- | --- | --- | --- |
| **Domain 1: Research team and reflexivity** | | |  | | |
| Personal Characteristics | | |  | | |
| 1. | Interviewer/facilitator | Which author/s conducted the interview or focus group? | Methods: pages 7 and 8 (Data collection), lines 24-25 and 1-3 | | |
| 2. | Credentials | What were the researcher's credentials? *E.g. PhD, MD* | Methods: pages 7 and 8 (Data collection), lines 24-25 and 1-3 | |  |
| 3. | Occupation | What was their occupation at the time of the study? | Methods: pages 7 and 8 (Data collection), lines 24-25 and 1-3 | |  |
| 4. | Gender | Was the researcher male or female? | Methods: pages 7 and 8 (Data collection), lines 24-25 and 1-3 | |  |
| 5. | Experience and training | What experience or training did the researcher have? | Methods: pages 7 and 8 (Data collection), lines 24-25 and 1-3 | |  |
| Relationship with participants | | |  | |  |
| 6. | Relationship established | Was a relationship established prior to study commencement? | NA | |  |
| 7. | Participant knowledge of the interviewer | What did the participants know about the researcher? e*.g. personal goals, reasons for doing the research* | NA | |  |
| 8. | Interviewer characteristics | What characteristics were reported about the interviewer/facilitator? e.g. *Bias, assumptions, reasons and interests in the research topic* | Methods: pages 8 and 9 (Reflexivity), lines 23-25 and 1-3 | |  |
| **Domain 2: study design** | | |  | |  |
| Theoretical framework | | |  | |  |
| 9. | Methodological orientation and Theory | What methodological orientation was stated to underpin the study? *e.g. grounded theory, discourse analysis, ethnography, phenomenology, content analysis* | Methods:  Page 5 and 6 (Intervention development and Theory of change), lines 21-24 and 1-8  Page 9 (Data analysis), lines 8-10 | |  |
| Participant selection | | |  | |  |
| 10. | Sampling | How were participants selected? *e.g. purposive, convenience, consecutive, snowball* | Methods: page 7 (Setting and sampling); lines 13-16 | |  |
| 11. | Method of approach | How were participants approached? e*.g. face-to-face, telephone, mail, email* | Methods: page 7 (Setting and sampling); lines 16-18 | |  |
| 12. | Sample size | How many participants were in the study? | Methods: page 7 (Data collection); lines 21-23 | |  |
| 13. | Non-participation | How many people refused to participate or dropped out? Reasons? | Methods: page 7 (Setting and sampling); lines 16-18 | |  |
| Setting | | |  | |  |
| 14. | Setting of data collection | Where was the data collected? e*.g. home, clinic, workplace* | Methods: page 8 (Data collection); lines 4-5 | |  |
| 15. | Presence of non-participants | Was anyone else present besides the participants and researchers? | Methods: page 8 (Data collection); lines 4-5 | |  |
| 16. | Description of sample | What are the important characteristics of the sample? *e.g. demographic data, date* | Results: page 10, Table 1 | |  |
| Data collection | | |  | |  |
| 17. | Interview guide | Were questions, prompts, guides provided by the authors? Was it pilot tested? | Methods: page 8 (Data collection); lines 5-12 | |  |
| 18. | Repeat interviews | Were repeat interviews carried out? If yes, how many? | NA | |  |
| 19. | Audio/visual recording | Did the research use audio or visual recording to collect the data? | Methods: page 9 (Data analysis), line 6 | |  |
| 20. | Field notes | Were field notes made during and/or after the interview or focus group? | No | |  |
| 21. | Duration | What was the duration of the interviews or focus group? | Methods: page 8 (Data collection); lines 11-12 | |  |
| 22. | Data saturation | Was data saturation discussed? | Methods: page 7 (Setting and sampling); lines 18-19 | |  |
| 23. | Transcripts returned | Were transcripts returned to participants for comment and/or correction? | NA | |  |
| **Domain 3: analysis and findings** | | |  | |  |
| Data analysis | | |  | |  |
| 24. | Number of data coders | How many data coders coded the data? | Methods: page 9 (Data analysis), line 8-11 | |  |
| 25. | Description of the coding tree | Did authors provide a description of the coding tree? | No | |  |
| 26. | Derivation of themes | Were themes identified in advance or derived from the data? | Methods: page 9 (Data analysis), line 11-12 | |  |
| 27. | Software | What software, if applicable, was used to manage the data? | Methods: page 9 (Data analysis), line 11-12 | |  |
| 28. | Participant checking | Did participants provide feedback on the findings? | No | |  |
| Reporting | | |  | |  |
| 29. | Quotations presented | Were participant quotations presented to illustrate the themes / findings? Was each quotation identified? e*.g. participant number* | Results: page 10-19 | |  |
| 30. | Data and findings consistent | Was there consistency between the data presented and the findings? | Yes | |  |
| 31. | Clarity of major themes | Were major themes clearly presented in the findings? | Yes | |  |
| 32. | Clarity of minor themes | Is there a description of diverse cases or discussion of minor themes? | Yes | |  |

Developed from: Tong A, Sainsbury P, Craig J. Consolidated criteria for reporting qualitative research (COREQ): a 32-item checklist for interviews and focus groups. *International Journal for Quality in Health Care*. 2007. Volume 19, Number 6: pp. 349 – 357
